# Evolution of Vertebrate GnRH Receptors from the Perspective of a Basal Vertebrate

**DOI:** 10.3389/fendo.2012.00140

**Published:** 2012-11-19

**Authors:** Stacia A. Sower, Wayne A. Decatur, Nerine T. Joseph, Mihael Freamat

**Affiliations:** ^1^Department of Molecular, Cellular and Biomedical Sciences, Center for Molecular and Comparative Endocrinology, University of New HampshireDurham, NH, USA

**Keywords:** gonadotropin-releasing hormone receptors, G protein-coupled receptors, evolution, lamprey, basal vertebrate, receptor, hormone, pituitary

## Abstract

This minireview provides the current status on gonadotropin-releasing hormone receptors (GnRH-R) in vertebrates, from the perspective of a basal vertebrate, the sea lamprey, and provides an evolutionary scheme based on the recent advance of whole genome sequencing. In addition, we provide a perspective on the functional divergence and evolution of the receptors. In this review we use the phylogenetic classification of vertebrate GnRH receptors that groups them into three clusters: type I (mammalian and non-mammalian), type II, and type III GnRH receptors. New findings show that the sea lamprey has two type III-like GnRH receptors and an ancestral type GnRH receptor that is more closely related to the type II-like receptors. These two novel GnRH receptors along with lGnRH-R-1 share similar structural features and amino acid motifs common to other known gnathostome type II/III receptors. Recent data analyses of the lamprey genome provide strong evidence that two whole rounds of genome duplication (2R) occurred prior to the gnathostome-agnathan split. Based on our current knowledge, it is proposed that lGnRH-R-1 evolved from an ancestor of the type II receptor following a vertebrate-shared genome duplication and that the two type III receptors resulted from a duplication within lamprey of a gene derived from a lineage shared by many vertebrates.

## Introduction

The study of gonadotropin-releasing hormone (GnRH) receptors in basal and later-evolved vertebrates can provide insight into the evolution and molecular mechanisms of signaling of this receptor family. Numerous full-length GnRH receptor (GnRH-R) sequences have been identified, with more than one receptor isoform identified within a single species. In most vertebrates there are usually two to three forms of GnRH-Rs present (Millar, 2005), although there are fewer GnRH-R genes in mammals compared to protochordates, fish, and amphibians (Morgan and Millar, 2004). To date, there is only a partial understanding of the physiological significance of each receptor type in regards to the spatial expression of GnRH-Rs since more than one receptor type can be expressed in the same tissue.

Of the numerous available forms of GnRH-R nomenclature that have been proposed by several investigators, in this review, we use the phylogenetic classification in which three distinct classes of GnRH-Rs from the vertebrate lineage group into separate clusters: type I (mammalian and non-mammalian), type II, and type III GnRH-Rs (Millar, 2005), with a new slight modification based on partial synteny analysis. Vertebrate type I GnRH-Rs are represented in teleosts, amphibians, reptiles, and avian species and in mammals. The type II GnRH-Rs include receptors from amphibians, reptiles, and mammals; however, type II GnRH-Rs are inactivated in most mammals studied to date (Stewart et al., 2009). Unlike the type II GnRH-Rs, the type III GnRH-Rs include sequences from teleost fish, amphibians, reptiles, and avian species but do not occur in mammals. New findings show that an ancestral extant vertebrate, the lamprey, has two type III-like GnRH-Rs and an ancestral type GnRH-R that is more closely related to the type II-like GnRH-Rs (Joseph et al., 2012). Based on our current knowledge, it is proposed that lGnRH-R-1 evolved from an ancestor of the type II receptor following a vertebrate-shared genome duplication and that the two type III receptors resulted from a duplication within lamprey of a gene derived from a lineage shared by many vertebrates.

Gonadotropin-releasing hormone action is mediated through high affinity binding with the GnRH receptor (GnRH-R), a rhodopsin-like seven transmembrane G protein-coupled receptor (GPCR). Pituitary GnRH receptors are thought to signal primarily through Gα_q/11_, resulting in the stimulation of the inositol phosphate (IP) second messenger system; however, Gα_s_ activation and cAMP signaling have been reported as well (Arora et al., 1995; Stanislaus et al., 1998; Grosse et al., 2000; Liu et al., 2002; Oh et al., 2005).

Lampreys along with hagfish are the only living representatives of the agnathans, the most ancient class of vertebrates, whose lineage dates back over 550 million years (Sower et al., 2009). Lampreys, which express three hypothalamic peptides of GnRH, lamprey GnRH-I, -II, and -III are important to our understanding of the reproductive endocrinology of the first vertebrates and are likely to have retained key characteristics of the ancestral GnRH and GnRH receptor from which modern GnRH isoforms and GnRH receptors arose, as reviewed (Sower et al., 2009).

## Molecular Evolution

As a basal vertebrate, the sea lamprey is well positioned to give us insight into the evolution of the GnRH receptors, particularly in reference to the seminal event of the acquisition of the hypothalamic-pituitary-gonadal axis in vertebrates (Sower et al., 2009). In framing this review, it is important to recognize that there are substantial factors acting in this immensely broad and profound transition that started approximately 550 million years ago with the beginnings of the vertebrate lineages. This shift was facilitated by two rounds of whole-genomic duplication (WGD) between the divergence of the protochordates and the lineage leading to modern vertebrates (Putnam et al., 2008; Van de Peer et al., 2010). Up until very recently, the placement of these two rounds of WGD relative to the split of the agnathans and gnathostomes was in question with the prevailing view placing the second round after the branching of the lamprey from the vertebrate lineage (see for example, Andreakis et al., 2011; Dores, 2011; Parmentier et al., 2011). Recent analysis of the numbers of paralogous gene regions and the pattern of shared synteny represented in the current sea lamprey genome assembly provides new evidence supporting two rounds of WGD occurring prior to the split of the agnathans and gnathostomes (Smith et al., submitted).

Recent identification and phylogenetic analysis of the three GnRH receptors in the sea lamprey (Joseph et al., 2012) in tandem with a reasonable-quality genome assembly (Smith et al., submitted) followed by more in-depth synteny analysis will help to elucidate the overall molecular evolution of the GnRH receptor family. Indeed, a similar approach has been most illuminating in the case of the family of vertebrate GnRHs. Shared synteny analysis of the genome (Decatur et al., 2011; Smith et al., submitted) clarified the ancestry of the genes for the lamprey GnRH peptides compared to the previous extensive phylogenetic studies that had been done (Guilgur et al., 2006; Kah et al., 2007; Kavanaugh et al., 2008; Okubo and Nagahama, 2008; Tsai and Zhang, 2008; Zhang et al., 2008). The synteny data provide evidence for an alternate view of the evolution of the GnRH peptide family and suggest that all duplication events that generated the different fish and tetrapod GnRH groups likely took place before the split of the ancestral lamprey and gnathostome lineages (Smith et al., submitted). The lamprey GnRH-I and -III, formerly referred to as group IV (Kavanaugh et al., 2008; Zhang et al., 2008) share a more recent common ancestry with GnRH2 and three paralogs (Decatur et al., 2011).

**Figure 1:**
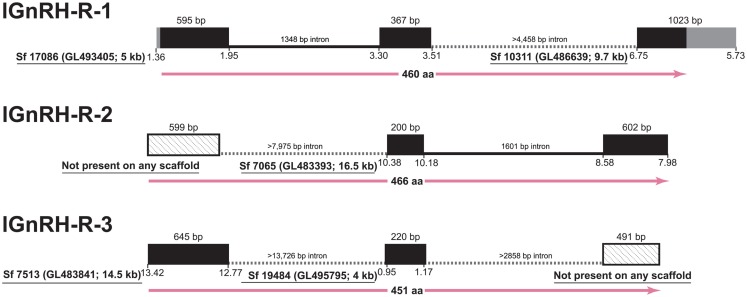
**Gene structure of the three GnRH receptors in lamprey in the context of the current gene assembly**. Exons are represented as black boxes with 5′- and 3′-UTRs in gray. In cases of incomplete data, angled-stippled fill is used for exons identified by cDNA sequencing that are not present among assembled scaffolds, and minimum lengths of the introns are provided based on the available flanking sequences of the scaffolds. Sf, Scaffold from Lamprey genome project consortium; details of the Ensembl scaffold designation are provided in parentheses, along with the size rounded to kilobase (kb). The position on the scaffold corresponding to the transcribed regions is below the boxes. At the bottom of each structure, the length of the deduced protein is given in amino acids with.

However, in the case of the GnRH receptors themselves, the state of the lamprey genome assembly precludes full analyses of the shared synteny, and genomic structure (Figure 1). Although half of the assembly is in scaffolds of 173 kb or longer (Smith et al., submitted), significant portions of the genome remain incompletely assembled and prevents investigations of many of the neuroendocrine genes in lamprey (Decatur et al., 2011). A similar issue was faced by Meyer and colleagues in their work resolving orthology of vertebrate genes (Qiu et al., 2011). These authors concluded that single exon genes are the best candidates to use given the current state of the assembly (Qiu et al., 2011). The genes for the lamprey GnRH peptides possess introns in the coding region except one (Kavanaugh et al., 2008) and the genes themselves encode small precursors (ca. 90 amino acids) that serendipitously fall on scaffolds of much larger size (95 and 302 kb) than those of the GnRH receptors. The open reading frame for the GnRH receptors in lamprey are substantially larger than those of the GnRH precursor and span three exons and two introns, similar to GnRH receptor genes of gnathostomes and even some genes of the protochordate amphioxus (Tello and Sherwood, 2009). A number of factors, including the high GC-content, vast numbers of repetitive elements, and dramatic genetic rearrangements that occur during somatic cell development, have been suggested as contributing to the challenge of sequencing and assembling a complete version of the lamprey genome (Smith et al., 2009, submitted).

**Figure 2:**
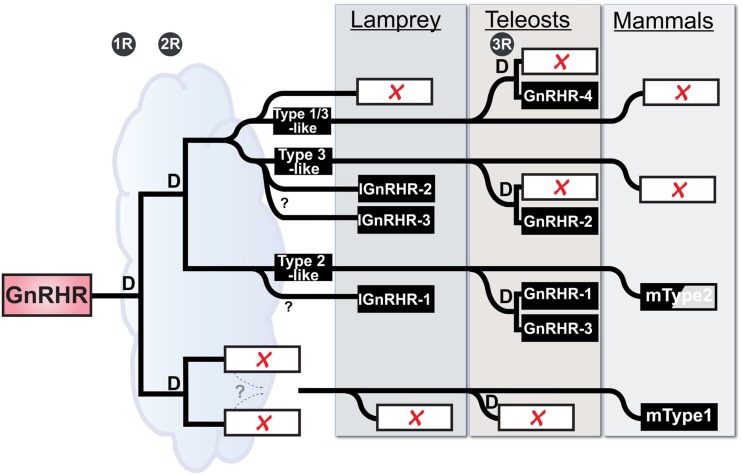
**Working Hypothesis on the evolution of the GnRH receptor gene family in vertebrates with emphasis on placement of the lamprey genes**. “D” represents duplication events for each of the 3 rounds of whole genome duplication in vertebrate evolution (1R, 2R, and 3R), the third being specific to teleosts. Open rectangles with red X’s indicate lost loci. Zebrafish are used here as a representative for teleosts. The half-shaded box for mammalian Type2 indicates that there is no functional gene product produced in several mammals, including humans. The cloud suggests the ambiguity concerning the relative time between early events, in particular the duration available for resolution of duplicated paralogs between the last common whole genome duplication event and the split of the agnathans from the gnathostome lineage. The dashed lines specify a proposed gene translocation, see Kim et al. (2011) for details and more vertebrates.

Accepting these limitations, we can use the initial characterization of the lamprey GnRH receptor evolution proposed by Kim et al. (2011) that was based on shared synteny and provide a new current model on GnRH receptor evolution based on the latest, although limited, synteny from the lamprey genome and our recent phylogenetic analysis (Figure 2). Maximum likelihood-based phylogenetic analysis showed a substantial degree of similarity (ca. 55% identity) between lamprey GnRH receptor 2 and 3 suggesting that these genes shared a recent ancestor. We further propose that they arose by local tandem or segmental duplication of one of the receptors within the lamprey lineage (Joseph et al., 2012). Based on this information, we expect lamprey GnRH receptors 2 and 3 are on the lineage shared by the zebrafish type III GnRH receptor, whereas lamprey GnRH receptor 1 is most similar to the lineage on which the mammalian type II receptor occurs. Teleost fish have undergone a third round (3R) of WGD. Following 3R, there was retention of some of these paralogs resulting in a greater number of GnRH receptor genes in teleosts (Kim et al., 2011).

## Functional Divergence of GnRH Receptors

As a measure of function, we looked at the binding characteristics of the GnRH receptors using GnRH ligands, and as a measure of receptor activation we looked at the signal transduction (Joseph et al., 2012). In an attempt to decipher the evolutionary lineage of specific motifs in terms of binding ability and signal transduction, we compared the three identified lamprey GnRH receptors (lGnRH-R-1, lGnRH-R-2, and lGnRH-R-3) with GnRH receptors of later vertebrates. Given the limited synteny data, we will describe this functional divergence of the GnRH receptors based solely in phylogenetic analysis, and not incorporate the emerging new current model on GnRH receptor evolution (described above). On the premise that lGnRH-R-1 evolved from a common ancestor of the type II GnRH receptor, we propose that identification of key motifs can assist in the elucidation of these motifs/residues in terms of evolutionary stringency. lGnRH-R-2 and lGnRH-R-3 which likely occurred due to a local gene duplication in the lamprey lineage may provide evidence of plasticity in amino acid residue functionality. The table below summarizes the key motifs of binding kinetics and signal transduction through studies on vertebrates in relation to those of an extant agnathan, the lamprey (Table 1).

**Table 1:** **Comparison of characteristics contributing to non-mammalian receptors and motifs pertaining to ligand binding and receptor activation of GnRH-R types**.

	Motif/residue	Mammalian (gnathostome)		Amphibian (gnathostome)			Petromyzonids (agnatha)		
Species		Human	Mouse	Bullfrog			Sea lamprey		
Receptor type		Type I	Type I	Type I	Type II	Type III	Type III	Type III	Ancestral type
Receptor abbreviation		hGnRH-R	mGnRH-R	bfGnRH-R-2	bfGnRH-R-3	bfGnRH-R-1	lGnRH-R-2	lGnRH-R-3	lGnRH-R −1
Non-mammalian characteristic	C-terminal tail Asp, helix 2, and helix 7 MD	No Asn in helix 2, Asp in helix 7	No Asn in helix 2, Asp in helix 7	Yes Asp in helix 2 and 7	Yes Asp in helix 2 and 7	Yes Asp in helix 2 and 7	Yes Asp in helix 2 and 7	Yes Asp in helix 2 and 7	Yes Asp in helix 2 and 7
Ligand binding	Asp, TMD 2	Asp	Asp	Asp	Asp	Asp	Asp	Asp	Asp
	Asn, TMD 2	Asn	Asn	Asn	Asn	Asn	Asn	Asn	His
	Lys, TMD 3	Lys	Lys	Lys	Lys	Lys	Lys	Lys	Arg
	“SE/DP” motif ECL 3	SDP	SEP	PEY	PPS	SQS	PEA	PEY	PHF
Gα_q/11_ coupling motifs	Leu, IL 2, and the Arg cage motif “D/ERY/XXXI/V”	Leu and DRSLAI	Leu and DRSLAI	Leu and DRHWAI	Leu and DRHAAI	Leu and DRQSAI	Leu and DRHAAV	Leu and DRYSAV	Leu and DRHSAI
	Ala, ICL3	Ala	Ala	Ala	Ala	Ala	Ala	Ala	Ala
	Leu, IL 3	Leu	Leu	Ile	Ile	Ile	Ile	Ile	Ile
cAMP	HFRK motif	(no C-tail)	(no C-tail)	SFKE	HFRR	HFRK	PLGP	QWDG	HVRR motif
	K71LSR75; L58;L80	KLSR; L; L	KLSR; L; L	CKSH; C; I	KKSH; W; I	KRSH; W; I	RRSH; W;L	RGSH; R;L	TKSH; C;I

*Representative sequences of non-mammalian type I GnRH-Rs (mouse and human) and amphibian type I, II, and III GnRH-Rs (bullfrog) were compared with the three identified GnRH receptors in the lamprey (Flanagan et al., 1994, 1999; Zhou et al., 1994, 1995; Arora et al., 1995, 1997; Davidson et al., 1996; Ballesteros et al., 1998; Myburgh et al., 1998; Chung et al., 1999; Fromme et al., 2001; Kitanovic et al., 2001; Millar et al., 2004; Wang et al., 2004; Li et al., 2005; Oh et al., 2005)*.

All three GnRH receptors in the lamprey have similar non-mammalian GnRH receptor characteristics (Zhou et al., 1994; Flanagan et al., 1999; Millar et al., 2004). However in terms of ligand binding, there is less conservation of key residues in lGnRH-R-1 compared to the two type III lGnRH receptors. Of the four ligand binding residues considered, two His residues are substituted in lGnRH-R-1 for Asn (Davidson et al., 1996) and Glu (Flanagan et al., 1994), suggesting there is less conservation of key residues in lGnRH-R-1 when compared to the type III lamprey GnRH receptors. However, lGnRH-R-1 remains as the only lamprey receptor displaying an affinity to lGnRH-I, despite all the lamprey receptors coding for Asp in the helix2/7 microdomain, enabling binding of configured and non-configured ligands (Zhou et al., 1994; Flanagan et al., 1999). Perhaps there are motifs retained within the ancestral type II-like lamprey GnRH receptor (lGnRH-R-1) that were stringently selected for in terms of binding affinity which would need to be determined by mutation analysis.

In relation to the examined signal transduction residues and motifs known to be responsible for Gα_q/11_ coupling and cAMP accumulation there are no striking substitutions between the three lamprey GnRH receptors except for the HFRK motif recognized for its attribution for cAMP signal transduction (Oh et al., 2005). lGnRH-R-1 has less substitutions at this motif when compared to the type III lGnRH-Rs and is the only lamprey GnRH receptor able to result in cAMP accumulation when activated by endogenous ligands (Kavanaugh et al., 2008; Joseph et al., 2012). lGnRH-R-1, which is proposed to have arisen from a common ancestor of type II receptors, retained this feature compared to the type III lamprey GnRH receptors that do not have this HRFK-like motif. It is difficult to attribute receptor types to specific signal transduction pathways, although it will be more feasible, when more representative receptors from each vertebrate are examined. However, it may remain that the plasticity in formation of signal transduction complexes drives the retention of specific GnRH receptor types.

Interestingly, given lGnRH-R-1 remains as the only receptor to bind to lGnRH-I (Joseph et al., 2012), investigation of evolutionary stringency of receptor residues/motifs in comparing lGnRH-R-1 (type II-like GnRH receptor) and other type II GnRH receptors suggests a selective co-evolution of cognate ligand/receptor pairing of lGnRH-I and lGnRH-R-1. However, in terms of plasticity in amino acid residue functionality, we surmise that the examined residues of the type III lGnRH receptors are pertinent for receptor functionality.

## Physiological Roles of GnRH Receptors

Physiological roles of GnRH receptor isoforms are the result of the combined effects of binding and activation of the receptor by one or multiple forms of GnRH at the molecular structural level and the spatio-temporal regulation of its expression in one or multiple tissues of an organism (True and Carroll, 2002). Major mutational events like whole genome duplications as well as local gene duplications generate new input for the evolutionary processes that result in creation of new morphological or functional characteristics. Functional divergence of protein sequences through neo- or sub-functionalization of protein sequences is one of the main mechanisms of the generation of evolutionary novelty. Consequently, the last decade has seen an increase in the number of research studies dedicated to detection of the traces of purifying or adaptive changes in the coding regions of various proteins as well as to understanding of co-evolutionary mechanisms that link receptors to their ligands. We briefly reviewed here the main sequence determinants of ligand binding and signal transduction in GnRH receptors and their conservation from the perspective of sea lamprey isoforms.

In contrast, much less attention has been given to the other source of adaptive potential generated by duplication events, i.e., the non-coding genomic regions. Investigation of GnRH receptors, of their expression pattern in relation to their role in reproductive or non-reproductive physiological processes has generated a wealth of experimental data regarding these aspects in numerous vertebrate taxa. An overview of these data suggests that the GnRH/GnRH receptor system is a very interesting system to be approached from the perspective of the role of gene expression regulation in its evolution, given the relative promiscuity of GnRH receptors and their wide spectrum of tissue expression (Chen and Fernald, 2008). This is however a considerably more difficult endeavor than modeling the coding sequence evolution. At the DNA level, the regions of particular interest may include not only the *cis*-acting control elements of the target gene but also the *cis* elements of the network of *trans*-acting factors that ultimately determine the expression not only of the receptor but also of its GnRH ligands (Fraser, 2011).

The strength of lamprey as an evolutionary model is that no other organism is better positioned to offer clues in respect to the earliest events in the evolution of the neuroendocrine mechanisms in vertebrates. It has evolved directly from the jawless ancestor of all vertebrates, independently from the gnathostome radiation. Its pituitary (hypophysis) is probably a plesiomorphic character in vertebrates and given the surprising overall morphological similarity of this animal with lamprey-like Devonian fossils (Janvier, 2006), it may also reflect the ancestral organization of this gland. Moreover, the experimental evidence accumulated so far on the function of lamprey pituitary is consistent with its involvement in the endocrine control of reproduction (Sower and Kawauchi, 2001; Sower et al., 2009).

As mentioned before, lamprey receptor isoforms are hypothesized to belong to two paralogous lineages shared with the gnathostomes (type II and III). A likely local duplication event within the type III lineage resulted in subsequent divergence of the lamprey-specific GnRH-R 2 and 3 pair of paralogs. Similarly with their orthologs in a majority of the gnathostome lineages, tissue expression of the lamprey receptors show a diverse, frequently overlapping pattern, connected to their three main functional roles in brain, pituitary, and gonadal physiology. The recently found lGnRH-R-2 precursor transcript was detected in the pituitary as well as in a wide variety of non-reproductive tissues. Interestingly, the expression control of the lGnRH-R-3 precursor transcript seems to have sharply diverged from its more recent paralog, its expression was detected only in the brain and eye of male and female lampreys (Joseph et al., 2012). In addition it seems to exhibit a sexually dimorphic expression pattern, being detected in the ovary but not in the testes. The previously described lGnRH-R-1 showed limited expression, its transcripts being detected only in the pituitary and testes of sea lamprey (Silver et al., 2005). A diverse and often overlapping functionality appears to characterize their interaction with lamprey GnRH isoforms as well: the widely expressed lGnRH-R-2 is activated by hypothalamic lGnRH-III and in a lesser extent by ubiquitous lGnRH-II. lGnRH-R-3 from brain and eyes has lGnRH-II as putative main activator and in a lesser extent lGnRH-III. lGnRH-R-1 in pituitary and gonads is promiscuously activated by GnRH-I, -II and -III but the interaction with hypothalamic GnRH-I seems to be highly selective. An interesting aspect of the evolutionary history of the lamprey isoforms is that their functional diversity was initiated by duplication events that are estimated to have taken place on different scales in the genome, i.e., local gene duplication (2 and 3) versus whole genome duplication (2, 3, and 1). Since the local duplications are more likely to change the regulatory environment of a gene and therefore to induce an immediate major shift in gene expression, this might offer the opportunity to compare the non-coding versus coding sequence roles in influencing the fate of paralogs.

## Summary

The accumulation of more data regarding the GnRH and GnRH receptors both in mammals as well as in more basal groups of vertebrates has started to unravel a more complex picture of their specificity of expression and ligand-receptor interaction. In turn, this leads to a more nuanced understanding of their physiological roles, both in relation to their central role in reproduction as well as outside the hypothalamic-pituitary system. Increase in the number of isoforms of vertebrate GnRH and GnRH receptors raises the question of their origin and of the evolutionary events and mechanisms that contributed to the situation seen in today’s vertebrates.

The constant in the convoluted history of GnRH receptor in vertebrates is the organization of the hypothalamo-pituitary axis. Different vertebrate lineages have solved the question of neural control of pituitary gonadotropin secretion using different pieces of the material generated by successive genome and/or gene duplications.

Understanding of the evolutionary mechanisms that molded the GnRH regulation of reproduction in vertebrates by acting at the level of protein expression requires further knowledge on genetic and epigenetic mechanisms and understanding the change in the endocrine, paracrine, and neural pathways that converge into regulating the release of the ligand from the GnRH neurons and expression of receptors in gonadotropes.

## Conflict of Interest Statement

The authors declare that the research was conducted in the absence of any commercial or financial relationships that could be construed as a potential conflict of interest.
